# Exosomes from Bone Marrow Mesenchymal Stem Cells with Overexpressed Nrf2 Inhibit Cardiac Fibrosis in Rats with Atrial Fibrillation

**DOI:** 10.1155/2022/2687807

**Published:** 2022-03-15

**Authors:** Lijuan Xu, Yingchao Fan, Liting Wu, Cui Zhang, Min Chu, Yuan Wang, Wenfang Zhuang

**Affiliations:** Medical Laboratory, Shidong Hospital, Yangpu District, Shidong Hospital Affiliated to University of Shanghai for Science and Technology, Shanghai 200438, China

## Abstract

**Background:**

Even though nuclear factor-erythroid 2-related factor 2 (Nrf2) signaling has been associated with the pathogenesis of multiple heart conditions, data on roles of Nrf2 within atrial fibrillation (AF) still remain scant. The present investigation had the aim of analyzing Nrf2-overexpressing role/s upon bone mesenchymal stem cell- (BMSC-) derived exosomes in rats with AF.

**Methods:**

Exosomes were collected from control or Nrf2 lentivirus-transduced BMSCs and then injected into rats with AF through the tail vein. AF duration was observed using electrocardiography. Immunohistochemical staining was then employed for assessing Nrf2, HO-1, *α*-SMA, collagen I, or TGF-*β*1 expression profiles within atrial myocardium tissues. Conversely, Masson staining was utilized to evaluate atrial fibrosis whereas apoptosis within myocardia was evaluated through TUNEL assays. In addition, TNF-*α*, IL-1*β*, IL-4, or IL-10 serum expression was assessed through ELISA.

**Results:**

Results of the current study showed significant downregulation of Nrf2/HO-1 within AF rat myocardia. It was found that injection of the control or Lv-Nrf2 exosomes significantly alleviated and lowered AF timespans together with reducing cardiomyocyte apoptosis. Moreover, injection of Lv-Nrf2 exosomes essentially lowered AF-driven atrial fibrosis and also inhibited inflammatory responses in the rats with AF.

**Conclusion:**

Delivery of BMSC-derived exosomes using overexpressed Nrf2 inhibited AF-induced arrhythmias, myocardial fibrosis, apoptosis, and inflammation via Nrf2/HO-1 pathway triggering.

## 1. Introduction

Atrial fibrillation (AF) is an irregular rhythm of the heart which is mainly characterized by arrhythmia, pulse disorder, palpitation, dizziness, chest discomfort, and shortness of breath [1]. Further, the main histological feature for AF is atrial fibrosis which is often caused by deposition of excessive fibrinogen due to dysregulation of extracellular matrix metabolism [1, 2]. Several previous studies have shown that apoptosis of atrial myocytes and atrial fibrosis lead to deceleration of atrial conduction velocity which mediates the occurrence and development of AF [3, 4].

Exosomes are vesicles discharged through multiple cell types and highly prevalent across bodily fluids. The exosomes have significant parts within cellular communications through DNA, RNA, and proteins [5, 6]. Exosomes have been widely utilized in the delivery of functional vectors to recipient cells. Besides, the exosomes have multiple pivotal parts within cellular signaling, cell differentiation, immune regulation, metabolic activities, and genomic expression control [7–9].

As vector delivery systems, exosomes were found to have pivotal parts within myocardial protection in cardiovascular diseases. For instance, after acute myocardial infarction, the exosomes obtained through adipose tissue-derived mesenchymal stem cells (AMSCs) upregulating sirtuin 1 (SIRT1) were found to reduce infarct size and atrial fibrotic area [10], whereas it is evident that the BMSC-derived exosomal microRNA-185 can inhibit ventricular remodeling in mice with myocardial infarction [11]. Further, a previous study conducted by Liu et al. [12] showed that AMSC-derived exosomal miR-320d inhibited apoptosis of myocardial cells in mice with AF.

However, data on the mechanisms of the exosomes in the treatment of AF has still remained elusive. Conversely, oxidative stress and inflammatory responses are thought to promote atrial fibrosis. Nuclear factor-erythroid 2-related factor 2 (Nrf2) is an essential transcription factor in cells [13, 14] and a key regulator of oxidative homeostasis. The Nrf2 remains continuously triggered throughout high oxidative stress, promoting transcription for target genes as well as translation of antioxidant and anti-inflammatory proteins [14, 15].

Previous studies demonstrated Nrf2 signaling to mediate the occurrence and development of a variety of heart diseases such as myocardial infarction, AF, or myocarditis [16–18]. However, data on whether Nrf2-overexpressed BMSC exosomes could block atrial fibrosis and thereby alleviate AF has still remained scant. Therefore, an AF rat model was constructed within this investigation, focusing upon Nrf2 influence within AF-specific atrial fibrosis and assess whether BMSC exosomes could serve as effective Nrf2 delivery systems in the alleviation of AF.

## 2. Materials and Methods

### 2.1. Animals

A total of 35 male Sprague-Dawley (SD) rats weighing between 230 and 250 g were purchased from Jiesijie Experimental Animal Company (Shanghai, China) and kept at 22-26°C, with between 40 and 60% humidity and under a 12-hour-12-hour light/dark cycle. Animals had unrestricted food/water supply. All in vivo assays were performed in line with directives and approvals from the animal ethics committee of Shidong Hospital Affiliated to University of Shanghai for Science and Technology.

### 2.2. BMSC Isolation

BMSCs were isolated from SD rats following the previous protocols [19]. Four-week-old rats were anesthetized through intraperitoneal injection of 1% pentobarbital (80 mg/kg) and soaked in 75% ethanol (15 min). Bilateral femurs/tibias were excised under aseptic environment and were consequently cleansed for between 3 and 5 times in 1X PBS containing penicillin (100 U/mL) and streptomycin (100 U/mL). After marrow cavity exposure, bone marrow was washed using serum-free DMEM/F12, followed by supernatant aspiration to prepare a single-cell suspension. This was placed into centrifugation (five minutes/1000 rpm) for cellular isolation. Pellets were washed with PBS followed by centrifugation and resuspension within 15 mL growth medium augmented with 10% fetal bovine serum (FBS) and 1% penicillin/streptomycin. Cellular suspension was transported into 25 cm^2^ culturing flasks and incubated at 37°C/5% CO_2_. Nonadherent cells were cultured in whole bone marrow adherent culture, and the culture medium was changed every 48 hours until cultures attained 80% confluency, whereby they underwent subculturing. Growth medium was discarded and the cells were washed twice in PBS. Eventually, cultures were trypsinized using 1 mL 2.5 g/L trypsin, and this cycle was repeated in order to attain the third passage of BMSCs required for subsequent assays.

### 2.3. BMSC Identification

BMSCs were introduced within 1.5 mL centrifuging tubes (10^5^ cells/tube) and then centrifuged for 5 minutes at 170 g. The supernatant was removed, followed by BMSC resuspension within 100 mL PBS. BMSCs were subjected to staining using FITC-labeled antibodies against CD29, CD90, or CD45 (Invitrogen™, USA) at room temperature (RT) (45 min/darkness). Following a triple wash using PBS, BMSCs were scrutinized using flow cytometry (FACScan, BD Biosciences).

### 2.4. BMSC Differentiation into Adipocytes/Osteoblasts

In order to obtain adipogenic/osteogenic differentiation, BMSCs were cultured as previously described, using adipogenic differentiation medium (Invitrogen) for 14 days and osteogenic differentiation medium (Invitrogen) for 21 days, respectively [20]. Cultures were consequently fixated through 10% formalin at 4°C. In order to visualize adipogenic differentiation, such cultures were subsequently treated with 0.3% Oil Red O staining solution (20 min/RT).

Further, fixed cells were stained using 2% Alizarin Red S (20 minutes/RT) for visualization of osteogenic differentiation. Cultures were triple washed using PBS, and imaging was performed through light microscopy (Olympus™, Japan).

### 2.5. Lentiviral Transduction

The lentiviral vector bears the Nrf2 (pHBLV-CMV-MCS-3FLAG-EF1-ZsGreen-T2A-PURO-Nrf2) or control vector (pHBLV-CMV-MCS-3FLAG-EF1-ZsGreen-T2A-PURO), with lentivirus development conducted through Hanbio Biotechnology™ (Hanheng Biotechnology Co., Ltd.™, China). Further, recombinant lentiviral vector or control vector, together with two auxiliary packaging plasmids (pSPAX2 and pMD2G), were cotransfected within HEK293T cultures for the lentiviral generation. The cells were cultured for 48 hours, and then, the supernatant was collected. The BMSCs were incubated using the lentiviral supernatant with initial multiplicity of infection (MOI) of 50. Transduction effectiveness was investigated following 48 h through Western blot.

### 2.6. Extraction, Identification, and Labeling of Exosomes

Exosomes derived from the BMSCs or BMSCs with Nrf2-overexpressed lentivirus (Lv-Nrf2 BMSCs) were isolated through the Total Exosome Isolation Kit (from cellular culturing media) (Invitrogen™, USA) following the specification provided by the manufacturer. The exosomes were resuspended in PBS and then spread evenly over a copper grid. Thereafter, the exosomes were negatively stained with 3% phosphotungstate (five minutes/RT) and consequently scrutinized through transmission electron microscopy (Libra 120®; Zeiss™, Germany).

In the Western blot assay, exosomal biomarkers (CD63, CD81, CD9, TSG101, and Alix) served as positive controls, while the endoplasmic reticulum protein calnexin served as the negative control. The dimension range together with exosomal levels within nanoparticles was evaluated through nanoparticle monitoring assessments (Nanosight NS300®, Malvern, France) according to instructions provided by the manufacturer. Moreover, for tracking experiments, the exosomes were PKH26 Red-labeled through the PKH26 Red Fluorescent Labeling Kit® (Sigma™, USA) depending upon kit protocols [21].

### 2.7. Induction of AF in the Rat Model and Groups

Animals were segregated in a random manner within either the healthy control group (*n* = 5) or the AF group (*n* = 30). Animals within the control group were intravenously subjected to physiological sodium chloride once per day (0.1 mL/100 g) through the tail vein for 7 consecutive days. Conversely, the rats in the AF group received an injection of calcium chloride-acetylcholine (CaCl2-Ach; CaCl_2_, 10 mg/mL; Ach, 66 *μ*g/mL) once daily for a week for preparing the AF rat model [22]. The presence of standard F-waves and the absence of P-wave monitored through electrocardiogram (ECG) monitoring were deemed as reflective of achievement in developing AF animal models, which were consequently segregated in a random manner within three groups (*n* = 10/group): AF+PBS, AF+control exosomes, or AF+Lv-Nrf2 exosomes. In the AF+PBS group, AF rats were treated with intramyocardial injection of PBS whereas the AF+control exosome group received intramyocardial injection of exosomes from BMSCs.

Rats in the AF+Lv-Nrf2 exosomes were treated with intramyocardial injection of exosomes from Lv-Nrf2 BMSCs. In addition, PKH26-labeled exosomes or PBS injection was performed once a week to the AF rats, whereas injection of CaCl_2_-Ach solution was performed every after 3 days for 3 weeks. Further, exosomes (100 *μ*g proteomic content) within 20 *μ*L PBS were injected within the free wall of the left ventricle. Within the present study, the duration of AF in each group was recorded on assay termination. Blood samples were extracted from the carotid artery after ECG monitoring and subjected to centrifugation (15 minutes/3500 g), with the supernatant consequently stored at -80°C for biochemical analysis. Conversely, myocardial tissue samples were collected either in 4% paraformaldehyde for tissue sectioning at room temperature or at -80°C for further biochemical and histological analyses. Finally, the exosomal distribution within the myocardium was then monitored under fluorescence microscopy (Nikon™, Japan).

### 2.8. Masson Staining

The hearts were sliced into coronal plane segments, fixed using 4% paraformaldehyde at 4°C, and dried within graded ethanol, followed by paraffin embedding to quantify atrial fibrosis. The sections were cut into 4 *μ*m thick slices and stained using Masson's trichrome, with subsequent imaging through the use of a digital camera under a microscope (Olympus™, Japan). ImageJ® software (National Institutes of Health, USA) was employed for quantifying fibrotic areas and reflected the percentage of the blue-positive stained region to the total tissue region.

### 2.9. Terminal Deoxynucleotidyl Transferase-Mediated dUTP Nick-End Labeling (TUNEL) Assay

Cellular apoptosis within myocardial tissues was assessed through TUNEL staining using a TUNEL Kit® (Roche™, Germany) in line with kit protocols. Brown-nucleated cells were deemed as TUNEL positive, whereby all TUNEL-positive cells within three randomized 200x visual fields were quantified under a microscope (Olympus). The observer was blinded to the treatment groups, and the TUNEL-positive cells were reflected as percentage of total cells.

### 2.10. Western Blot Analysis

The BMSCs, exosomes, and myocardial tissue were subjected to lysis through RIPA lysis buffer® (Millipore Corporation™, Billerica, MA, USA), with the total proteomic concentration in cell exosomes or tissue asserted using a BCA protein assay kit® (Beyotime™, Shanghai, China), following the protocol provided by the manufacturer. Equivalent proteomic content was resolved in 10% SDS-PAGE gels and transferred into PVDF membranes (Merck Millipore™, MA, USA) which were blocked using 5% nonfat milk for 60 min/RT.

The membranes were incubated using primary antibodies against Nrf2 (1 : 1000, Invitrogen), HO-1 (1 : 1000, Abcam, Cambridge, UK), CD9 (1 : 500, Invitrogen), CD63 (1 : 500, Invitrogen), CD81 (1 : 2000, Abcam), TSG101 (1 : 500, Santa Cruz, CA, USA), Alix (1 : 500, Santa Cruz), calnexin (1 : 500, Santa Cruz), or GAPDH (1 : 10000, Abcam) at 4°C overnight, followed by incubation using HRP-linked goat anti-rabbit IgG (1 : 5000, Abcam) at 37°C for 1 hour. Proteomic bands were developed and scrutinized through enhanced chemiluminescence (ECL) (Thermo Scientific™, Rockford, IL, USA), quantified using ImageJ software.

### 2.11. Immunohistochemistry

Paraffin-embedded myocardial tissues in each group were sliced into 4 *μ*m thick segments, which were consequently deparaffinized using xylene (Sigma) and then treated using 3% H_2_O_2_ for quenching endogenous peroxidase action. Segments were then incubated (30 min) using citrate buffer within a steamer for mediating antigen retrieval. Thereafter, the segments were stained overnight using primary antibodies against Nrf2 (1 : 100, Invitrogen), HO-1 (1 : 500, Abcam), *α*-SMA (1 : 1000, Abcam), collagen I (1 : 500, Abcam), or TGF-*β*1 (1 : 500, Abcam) at 4°C, with a subsequent 45-minute incubation period using goat anti-rabbit IgG (1 : 1000, Abcam) at RT. The DAB was consequently employed to identify specific protein within such samples, followed by rinsing/counterstaining using hematoxylin (30 s). Finally, the samples were scrutinized under a microscope (Olympus) and evaluated through ImageJ® software.

### 2.12. Enzyme-Linked Immunosorbent Assay (ELISA) Analysis

TNF-*α*, IL-1*β*, IL-4, or IL-10 serum levels were determined in the present study using ELISA kits (R&D Systems, Minneapolis, MN, USA), in line with kit protocols.

### 2.13. Statistical Analyses

All data obtained in the current study reflected means ± standard deviation (SD), and statistical analyses were conducted through GraphPad Prism® V6.01. One-way analysis of variance (ANOVA) with subsequent Tukey's post hoc multiple comparison tests was also conducted for determining statistical significance of variations among the groups. Furthermore, a *P* value < 0.05 was deemed to confer statistical significance.

## 3. Results

### 3.1. Characterization of Isolated Rat BMSCs

**Figure 1:**
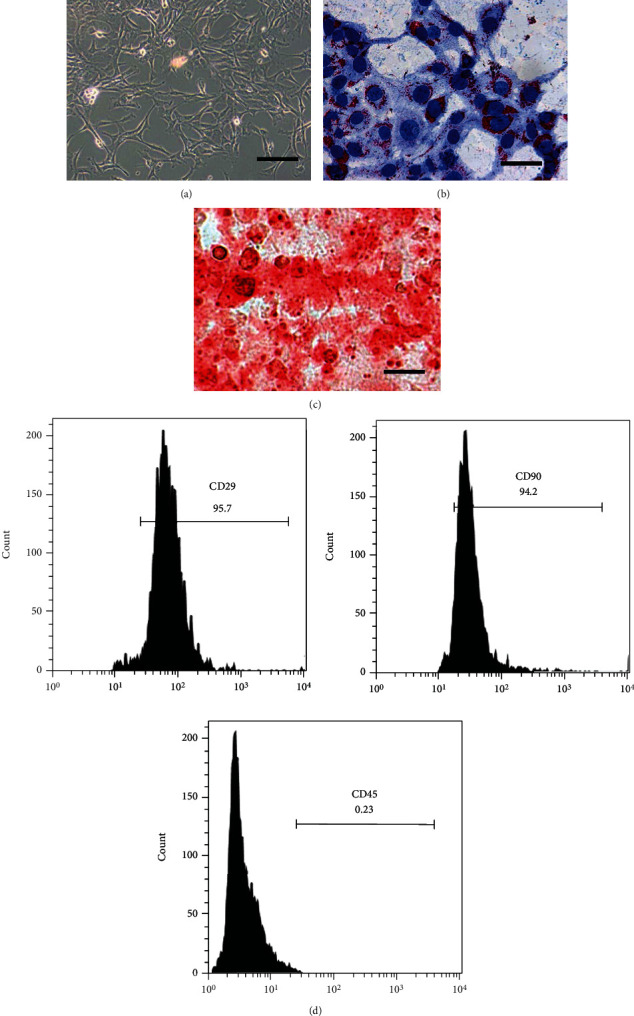
BMSC isolation and determination. (a) Cell morphology of cultured BMSCs. Scale = 100 *μ*m. (b) Adipocytes stained with Oil Red O. Scale bar = 50 *μ*m. (c) Osteoblasts stained with Alizarin Red. Scale = 50 *μ*m. (d) CD29, CD90, and CD45 expression as determined using flow cytometry.

The primary BMSCs obtained from the 4-week-old male SD rats showed a spindle-like morphology after 3 passages (Figure 1(a)). In order to further characterize BMSCs, their differentiation capacity was also tested. Following adipogenic induction for two weeks, it was found that lipid droplets concentrated around the nucleus (Figure 1(b)). A positive calcified nodule was distinctly noted following Alizarin Red S staining after osteogenesis induction for three weeks (Figure 1(c)). BMSC biomarkers were confirmed through flow cytometry, which was positive for CD29 and CD90 expression but negative for CD45 expression (Figure 1(d)). Therefore, the early characterizations identified a pure population of BMSCs that was successfully isolated from the rat.

### 3.2. The Isolation and Characterization of Exosomes

**Figure 2:**
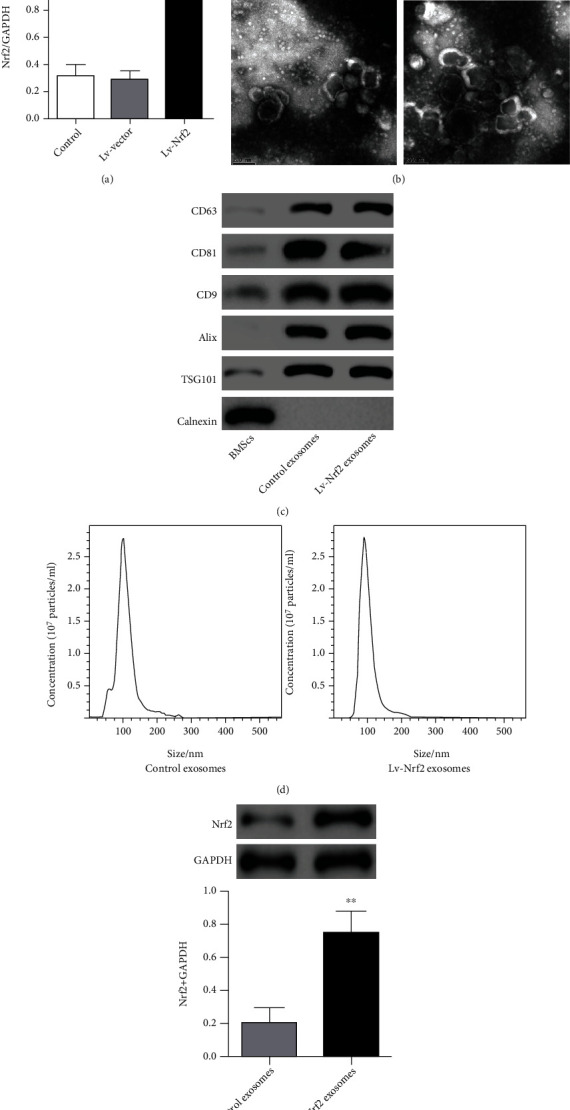
Characterization of exosomes derived from BMSCs. (a) BMSCs were transduced by lentivirus with overexpressed Nrf2 (Lv-Nrf2) for 48 h. Nrf2 expression was quantified by Western blot, where GAPDH served as the loading control. ^∗∗^*P* < 0.01 compared to control or Lv-vector groups. (b) Exosomes derived from the control BMSCs/Lv-Nrf2 BMSCs were analyzed by transmission electron microscopy. Scale = 200 nm. (c) Specific exosomal surface biomarker (positive markers: CD63, CD81, CD9, Alix, and TSG101; negative marker: calnexin) expression in the BMSCs and their exosomes was detected by Western blotting. (d) Diameter/concentration of exosomes was determined through nanoparticle tracking analysis. (e) Exosomal Nrf2 expression was determined through Western blot. ^∗∗^*P* < 0.01 compared to the control exosome group.

The present study used lentiviral vector (Lv-vector) or lentiviral Nrf2 overexpression (Lv-Nrf2) plasmids to transfect the BMSCs. Results of the current analysis showed that the BMSCs with the Lv-Nrf2 showed significant upregulation of the Nrf2 as compared with nontransfected control or Lv-vector-transfected BMSCs (Figure 2(a)). Exosomes were isolated from the BMSCs or BMSCs bearing Lv-Nrf2 and consequently profiled through transmission electron microscopy and Western blot. Results from the electron micrographs highlighted samples to have spheroid morphology, and overexpression of Nrf2 did not affect the exosome structure (Figure 2(b)).

Conversely, results of Western blot analysis showed high expression of exosomal surface markers (CD63, CD81, CD9, Alix, and TSG101) together with lack of the reticular marker calnexin in the exosomes isolated from both BMSCs (control exosomes) and BMSCs bearing Lv-Nrf2 (Lv-Nrf2 exosomes) (Figure 2(c)). Further, the results of NTA illustrated the size of the control exosomes and Lv-Nrf2 exosomes with a mean diameter of 100 nm (Figure 2(d)). In addition, Lv-Nrf2 exosomes showed increased Nrf2 protein expression as compared with the control exosomes (Figure 2(e)).

### 3.3. Lv-Nrf2 Exosomes Inhibited Heart Rhythm Changes in Rats with AF

**Figure 3:**
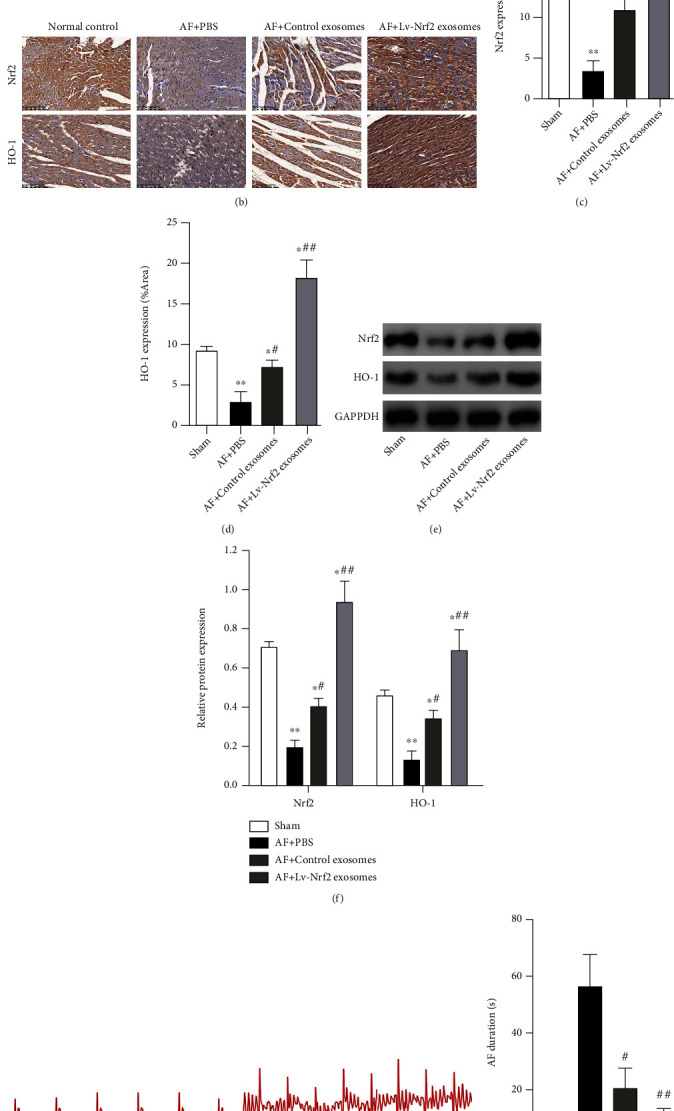
Lv-Nrf2 exosomes thwarted cardiac arrhythmias within AF rats. AF was established in rats using calcium chloride-acetylcholine (CaCl_2_-Ach) mixture via tail vein injection once per day, for seven consecutive days. The normal control group was intravenously treated with equivalent volume of 0.9% saline through the tail vein. Then, the exosomes derived from the control BMSCs or Lv-Nrf2 BMSCs were injected once a week to AF rats, and the CaCl_2_-Ach solution was continued to be injected into the tail vein every 3 days for 3 weeks, while the AF control group was treated with the equivalent level of PBS. (a) PKH26-labeled exosome distribution within the myocardium. Scale = 50 *μ*m. (b) The representative immunohistochemical stain showing the Nrf2/HO-1 cardiac expression within rats. Scale = 100 *μ*m. (c, d) Quantification for percentage positive Nrf2 (c)/HO-1 (d) staining area. (e) Representative Western blot imaging for Nrf2/HO-1 proteins involved within rat myocardial tissue across different groups. (f) Densitometric quantification showing the (e) relative proteomic expression normalized against GAPDH. (g) Changes in the electrocardiogram of each group were detected. (h) AF duration of each group was measured. *n* = 5 in each group. Results are presented as mean ± SD. ^∗^*P* < 0.05, ^∗∗^*P* < 0.01 in comparison to the control group. ^#^*P* < 0.05, ^##^*P* < 0.01 in comparison to the AF+PBS group.

In order to probe biological roles for exosomes from the Lv-Nrf2 BMSCs on the AF rat models, exosomes derived from the control BMSCs or Lv-Nrf2 BMSCs were also injected once a week to the AF rats for 3 weeks. The PKH26-labeled exosomes were observed within the myocardium for both control exosomes and Lv-N5rf2 exosomes but not in the normal control and PBS-treated groups (Figure 3(a)). Therefore, immunohistochemical staining and Western blot were then employed for assessing Nrf2 or HO-1 proteomic expression within myocardium samples from each group (Figures 3(b) and 3(e)).

The expression of both Nrf2 and HO-1 proteins was markedly suppressed in PBS-treated AF rats in comparison to the healthy control group. Expectedly, the Nrf2 and HO-1 proteins were upregulated in rats injected with control exosomes or Lv-Nrf2 exosomes, and both the Nrf2 and HO-1 levels were increased (Figures 3(c)–3(f)). The ECG for rats within the AF group demonstrated typical AF features, including the presence of standard F-waves and lack of P-wave, together with irregular RR intervals. In addition, such investigational outcomes demonstrated that rats in the normal control group exhibited sinus rhythms with similar P-waves/RR durations. Notably, injection of control or Lv-Nrf2 exosomes essentially blocked AF rat cardiac arrhythmias (Figure 3(g)). Furthermore, it was found that the AF-induced rats experienced extended duration of AF, while control exosomes or Lv-Nrf2 exosomes had shortened AF duration (Figure 3(h)).

### 3.4. Lv-Nrf2 Exosomes Suppressed the Expression of Fibrosis-Related Markers in AF Rats

**Figure 4:**
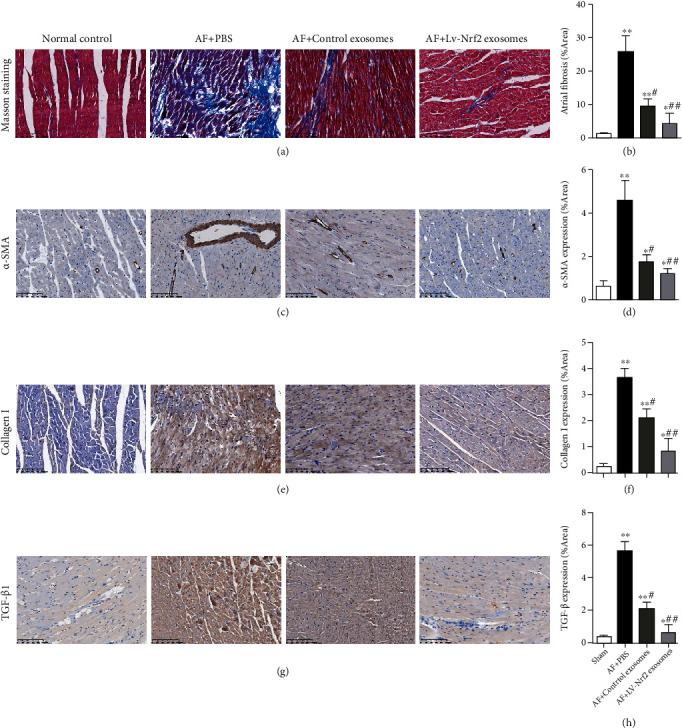
Lv-Nrf2 exosomes suppressed the expression of fibrosis-related markers in AF rats. (a) Masson stains for evaluating left atrial fibrosis within rats from each group. Scale = 100 *μ*m. (b) Quantification for the atrial fibrosis region. (c–h) The representative immunohistochemical staining and quantitative analysis showing the expression of atrial fibrosis makers *α*-SMA (c, d), collage I (e, f), or TGF-*β*1 (g, h) in the heart of the rats. Scale = 100 *μ*m. *n* = 5/group. Results reflected mean ± SD. ^∗^*P* < 0.05, ^∗∗^*P* < 0.01 in comparison to the control group. ^#^*P* < 0.05, ^##^*P* < 0.01 in comparison to the AF+PBS group.

To explore the effect of exosomes from the BMSCs overexpressing Nrf2 in atrial fibrosis induced by AF, collagen expression in the left atrial tissue of the rats was analyzed by Masson staining. Results from the Masson staining demonstrated high deposition of collagen in the left atrium and an increased area of myocardial fibrosis in the heart of PBS-treated AF rats in comparison to healthy control rats (Figures 4(a) and 4(b)). Besides, AF rats treated with control or Lv-Nrf2 exosomes exhibited less collagen deposition as compared with those from the PBS-treated group. In addition, treatment with Lv-Nrf2 exosomes showed less collagen content in the AF rats in comparison to content within the control exosome-treated group.

The expression of fibrosis-linked markers within left atrial rat tissue was analyzed by immunohistochemical staining (Figures 4(c)–4(h)). It was found that the protein expression of fibrosis-related markers *α*-SMA, collagen I, or TGF-*β*1 within atrial tissue was highly upregulated within AF rats having PBS treatment, with injection of control exosomes or Lv-Nrf2 exosomes suppressing the levels of protein expression. In addition, *α*-SMA, collagen I, or TGF-*β*1 expression within the Lv-Nrf2 exosome-treated AF rats was dramatically decreased in comparison to that within the control exosome-treated group.

### 3.5. Lv-Nrf2 Exosome Treatment Led to Reduction of Apoptotic Cells and Inflammation in Myocardial Tissue of AF Rats

**Figure 5:**
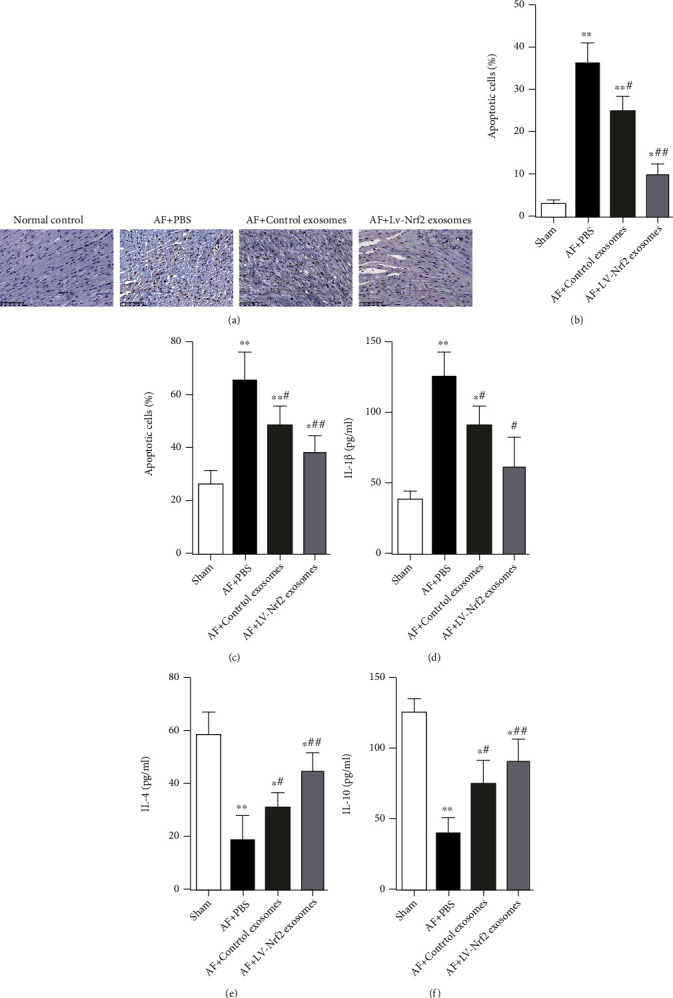
Lv-Nrf2 exosome treatment decreased apoptotic cells and inflammation in myocardial tissue of AF rats. (a, b) The degree of apoptosis in rat myocardia for every group was confirmed through TUNEL staining and quantitative analysis; scale bar = 100 *μ*m. (c–f) TNF-*α*, IL-1*β*, IL-4, or IL-10 expression in the rat serum was assessed via ELISA. *n* = 5 in each group. Results reflected mean ± SD. ^∗^*P* < 0.05, ^∗∗^*P* < 0.01 in comparison to the control group. ^#^*P* < 0.05, ^##^*P* < 0.01 in comparison to the AF+PBS group.

The TUNEL assay was used in the present study to quantify apoptosis of rat myocardial tissues in each group (Figures 5(a) and 5(b)). Results of the study showed a marked increase in apoptotic TUNEL-positive cells within PBS-treated AF rats as compared with the normal control group, whereas control or Lv-Nrf2 exosome-treated cells were linked to a major reduction in TUNEL-positive cell quantity. Further, the assessment of the expression of TNF-*α*, IL-1*β*, IL-4, or IL-10 by ELISA showed that control exosomes or Lv-Nrf2 exosomes markedly reduced AF-driven upregulation of TNF-*α* or IL-1*β* expression whereas downregulating IL-4 and IL-10 expression (Figures 5(c)–5(f)).

## 4. Discussion

Atrial fibrillation (AF) represents a highly prevalent arrhythmia [1]. Most of the patients with AF are prone to recurrent attacks after rhythmic transformation, which may lead to permanent AF and hence induce cardiac insufficiency as well as embolic diseases [23, 24]. Therefore, there is urgent need to explore pathogenesis of AF and develop new therapies to prevent and treat AF.

Previous investigations highlighted that exosomes could act as messengers for intercellular communications in cardiovascular diseases and cell-derived exosomes can serve as a potential alternative to stem cell therapy that mediates heart regeneration representing a rising niche for assessment and therapy of cardiovascular diseases [25–27]. In addition, previous studies have shown that exosomes derived from mesenchymal stem cells (MSCs) could inhibit myocardial inflammation, fibrosis, and apoptosis in AF mice [12]. In addition to MSC exosomes, exosomes secreted from stem cells of different origins and those derived from atrial myocytes [28] as well as myofibroblasts [29] have an effect on AF.

The current study showed that exosomes from BMSCs protect rats against AF-associated arrhythmias, myocardial fibrosis, apoptosis, and inflammation. Therefore, the BMSC-derived exosomes could constitute a viable treatment approach for AF. The protective effect of exosomes from different cells may be related to cytoprotective factors [30, 31]. For instance, Wang et al. [32] showed that MSC exosomes carrying microRNA-671 inhibit cardiomyocyte apoptosis, myocardial fibrosis, and inflammation by inactivating the TGFBR2/Smad2 axis.

Further, the present study also showed suppression of the Nrf2 and downstream HO-1 in myocardial tissue of rats with AF, causing elevated myocardial fibrosis, apoptosis, and inflammation. Besides, it was demonstrated that delivery of exosomes from the BMSCs overexpressing Nrf2 increased the Nrf2 and HO-1 expression and hence effectively inhibited changes in heart rhythm and shortened the AF duration, confirming that exosomes from Lv-Nrf2 BMSCs promoted recovery of arrhythmias in AF rats.

Cardiac remodeling is the main AF feature, defined through increasing oxidative stress [16, 33]. The Nrf2/HO-1 is a powerful antioxidant network that could contribute to prophylaxis against multiple oxidative stress-linked conditions [34–36]. For instance, according to Dong et al. [37], silencing of Nrf2 expression in H9C2 cells led to insufficient production of antioxidant enzymes by aldehyde for reversing oxidative damage led by tert-butyl hydrogen peroxide (TBHP) and hence greatly increases the chances of arrhythmia. However, myocardial oxidative damage can be alleviated through triggering the Nrf2 signaling pathway and blocking the generation of oxidation together with free radicals.

In addition, an investigation performed by Yeh and colleagues [38] demonstrated that statins protect AF by inhibiting myocardial remodeling through enhancing the expression of Nrf2/HO-1 within cardiomyocytes and reducing oxidative stress as well as myocardial fibrillation which is induced by tachycardia. Therefore, the Nrf2/HO-1 pathway has a pivotal part within myocardial protection for treating AF. It has been found that atrial fibrosis is associated with the occurrence of arrhythmias and the formation of atrial fibrosis results from excessive proliferation of a-SMA-labeled myofibroblasts and excessive deposition of extracellular matrix components, including type I collagen and TGF-*β*1 [39–41].

Results of analysis in the current study showed significant increase in the area of myocardial fibrosis and expression of *α*-SMA, type I collagen, and TGF-*β*1 in AF rats. However, exosomes from Lv-Nrf2 BMSCs significantly reduced the fibrotic areas and levels of *α*-SMA, type I collagen, and TGF-*β*1 proteins. These results indicate that exosomes from Lv-Nrf2 BMSCs could inhibit atrial fibrosis and hyperproliferation of myofibroblasts in AF rats.

Similarly, it has been reported that cardiac myocyte apoptosis occurs in patients with AF and apoptosis increases with the increase in duration as well as intensity of AF [42, 43]. Within this investigation, influence of BMSC exosomal Nrf2 upon cardiac apoptosis in AF rats was assessed through TUNEL staining. Results of the current study revealed that treatment with Lv-Nrf2 exosomes significantly reduced cardiac tissue apoptosis as compared with that in AF rats administered with PBS or control exosomes. Conversely, past investigations demonstrated that inflammation responses play a key role within progression of AF [44–46]. In agreement, it was evident that injection of BMSCs also controls exosomes and Lv-Nrf2 exosomes; in particular, Lv-Nrf2 exosomes markedly reduced AF-driven upregulation of TNF-*α* and IL-1*β* as well as downregulation of IL-4 and IL-10 in serum. The results of this investigation hence demonstrated the role of Lv-Nrf2 exosomes for inhibiting inflammatory response in AF rats.

## 5. Conclusion

In essence, this investigation highlighted Nrf2 and HO-1 downregulation within myocardial tissue of AF rats. Further, it was found that the delivery of Lv-Nrf2 exosomes inhibited AF-induced arrhythmias, myocardial fibrosis, apoptosis, and inflammation through Nrf2/HO-1 pathway triggering. Therefore, this study contributes a potential approach for treating AF through exosome-mediated intercellular communication.

## Data Availability

The datasets used and analyzed during the current study are available from the corresponding author on reasonable request.

## References

[1] Beyer C., Tokarska L., Stühlinger M. (2021). Structural cardiac remodeling in atrial fibrillation. *JACC Cardiovascular Imaging*.

[2] Paliwal N., Ali R. L., Salvador M. (2021). Presence of left atrial fibrosis may contribute to aberrant hemodynamics and increased risk of stroke in atrial fibrillation patients. *Frontiers in Physiology*.

[3] Wu N., Li C., Xu B. (2021). Circular RNA mmu_circ_0005019 inhibits fibrosis of cardiac fibroblasts and reverses electrical remodeling of cardiomyocytes. *BMC Cardiovascular Disorders*.

[4] Ma S., Ma J., Tu Q., Zheng C., Chen Q., Lv W. (2020). Isoproterenol increases left atrial fibrosis and susceptibility to atrial fibrillation by inducing atrial ischemic infarction in rats. *Frontiers in Pharmacology*.

[5] Sharma S., Masud M. K., Kaneti Y. V. (2021). Extracellular vesicle nanoarchitectonics for novel drug delivery applications. *Small*.

[6] Tutuianu R., Rosca A. M., Iacomi D. M., Simionescu M., Titorencu I. (2021). Human mesenchymal stromal cell-derived exosomes promote in vitro wound healing by modulating the biological properties of skin keratinocytes and fibroblasts and stimulating angiogenesis. *International Journal of Molecular Sciences*.

[7] Li Q., Wang H., Peng H., Huyan T., Cacalano N. A. (2019). Exosomes: versatile nano mediators of immune regulation. *Cancers*.

[8] Cui X., Liu Y., Wang S. (2019). Circulating exosomes activate dendritic cells and induce unbalanced CD4+ T cell differentiation in Hashimoto thyroiditis. *The Journal of Clinical Endocrinology and Metabolism*.

[9] Clark D. J., Fondrie W. E., Yang A., Mao L. (2016). Triple SILAC quantitative proteomic analysis reveals differential abundance of cell signaling proteins between normal and lung cancer-derived exosomes. *Journal of Proteomics*.

[10] Huang H., Xu Z., Qi Y. (2020). Exosomes from SIRT1-overexpressing ADSCs restore cardiac function by improving angiogenic function of EPCs. *Molecular Therapy Nucleic acids*.

[11] Li Y., Zhou J., Zhang O. (2020). Bone marrow mesenchymal stem cells-derived exosomal microRNA-185 represses ventricular remolding of mice with myocardial infarction by inhibiting SOCS2. *International Immunopharmacology*.

[12] Liu L., Zhang H., Mao H., Li X., Hu Y. (2019). Exosomal miR-320d derived from adipose tissue-derived MSCs inhibits apoptosis in cardiomyocytes with atrial fibrillation (AF). *Artificial cells, Nanomedicine, and Biotechnology*.

[13] Cho H. Y., Marzec J., Kleeberger S. R. (2015). Functional polymorphisms in *Nrf2*: implications for human disease. *Free Radical Biology & Medicine*.

[14] Cui T., Lai Y., Janicki J. S., Wang X. (2016). Nuclear factor erythroid-2 related factor 2 (Nrf2)-mediated protein quality control in cardiomyocytes. *Frontiers in Bioscience*.

[15] Saha S., Buttari B., Panieri E., Profumo E., Saso L. (2020). An overview of Nrf2 signaling pathway and its role in inflammation. *Molecules*.

[16] Wu X., Huang L., Liu J. (2021). Relationship between oxidative stress and nuclear factor-erythroid-2-related factor 2 signaling in diabetic cardiomyopathy (review). *Experimental and Therapeutic Medicine*.

[17] Vashi R., Patel B. M. (2021). NRF2 in cardiovascular diseases: a ray of hope!. *Journal of Cardiovascular Translational Research*.

[18] Yarmohammadi F., Hayes A. W., Karimi G. (2021). The cardioprotective effects of hydrogen sulfide by targeting endoplasmic reticulum stress and the Nrf2 signaling pathway: a review. *Bio Factors*.

[19] Yu T., Zhao C., Hou S., Zhou W., Wang B., Chen Y. (2019). Exosomes secreted from miRNA-29b-modified mesenchymal stem cells repaired spinal cord injury in rats. *Brazilian Journal of Medical and Biological Research*.

[20] Wang Z., Lin Y., Jin S., Wei T., Zheng Z., Chen W. (2020). Bone marrow mesenchymal stem cells improve thymus and spleen function of aging rats through affecting P21/PCNA and suppressing oxidative stress. *Aging*.

[21] Kim H., Kang J. Y., Mun D., Yun N., Joung B. (2019). Calcium chloride enhances the delivery of exosomes. *PLoS One*.

[22] Li Y., Song B., Xu C. (2018). Effects of Guanfu total base on Bcl-2 and Bax expression and correlation with atrial fibrillation. *Hellenike Kardiologike Epitheorese*.

[23] Grymonprez M., Steurbaut S., De Sutter A., Lahousse L. (2020). Impact of a single non-sex-related stroke risk factor on atrial fibrillation and oral anticoagulant outcomes: a systematic review and meta-analysis. *Open Heart*.

[24] Wei W., Shehata M., Wang X. (2019). Invasive therapies for patients with concomitant heart failure and atrial fibrillation. *Heart failure Reviews*.

[25] Ong S. G., Wu J. C. (2015). Exosomes as potential alternatives to stem cell therapy in mediating cardiac regeneration. *Circulation Research*.

[26] Wang H., Xie Y., Salvador A. M. (2020). Exosomes: multifaceted messengers in atherosclerosis. *Current Atherosclerosis Reports*.

[27] Germena G., Hinkel R. (2021). iPSCs and exosomes: partners in crime fighting cardiovascular diseases. *Journal of Personalized Medicine*.

[28] Li J., Zhang Q., Jiao H. (2021). LncRNA NRON promotes M2 macrophage polarization and alleviates atrial fibrosis through suppressing exosomal miR-23a derived from atrial myocytes. *Journal of the Formosan Medical Association*.

[29] Li S., Gao Y., Liu Y. (2020). Myofibroblast-derived exosomes contribute to development of a susceptible substrate for atrial fibrillation. *Cardiology*.

[30] Qi Z., Wu D., Li M. (2020). The pluripotent role of exosomes in mediating non-coding RNA in ventricular remodeling after myocardial infarction. *Life sciences.*.

[31] Wang X., Chen Y., Zhao Z. (2018). Engineered exosomes with ischemic myocardium-targeting peptide for targeted therapy in myocardial infarction. *Journal of the American Heart Association.*.

[32] Wang X., Zhu Y., Wu C., Liu W., He Y., Yang Q. (2021). Adipose-derived mesenchymal stem cells-derived exosomes carry microRNA-671 to alleviate myocardial infarction through inactivating the TGFBR2/Smad2 axis. *Inflammation*.

[33] Wolke C., Bukowska A., Goette A., Lendeckel U. (2015). Redox control of cardiac remodeling in atrial fibrillation. *Biochimica et Biophysica Acta*.

[34] Zhang X., Yu Y., Lei H. (2020). The Nrf-2/HO-1 signaling axis: a ray of hope in cardiovascular diseases. *Cardiology Research and Practice*.

[35] Pol O. (2021). The role of carbon monoxide, heme oxygenase 1, and the Nrf2 transcription factor in the modulation of chronic pain and their interactions with opioids and cannabinoids. *Medicinal Research Reviews*.

[36] Ucar B. I., Ucar G., Saha S., Buttari B., Profumo E., Saso L. (2021). Pharmacological protection against ischemia-reperfusion injury by regulating the Nrf2-Keap1-ARE signaling pathway. *Antioxidants*.

[37] Dong J., Feng X., Zhang J. (2019). *ω*-3 fish oil fat emulsion preconditioning mitigates myocardial oxidative damage in rats through aldehydes stress. *Biomedicine & Pharmacotherapy*.

[38] Yeh Y. H., Kuo C. T., Chang G. J. (2015). Rosuvastatin suppresses atrial tachycardia-induced cellular remodeling via Akt/Nrf2/heme oxygenase-1 pathway. *Journal of Molecular and Cellular Cardiology*.

[39] Qiu H., Wu H., Ma J. (2018). DL-3-n-butylphthalide reduces atrial fibrillation susceptibility by inhibiting atrial structural remodeling in rats with heart failure. *Naunyn-Schmiedeberg's Archives of Pharmacology*.

[40] Ma R. F., Chen G., Li H. Z. (2020). Panax Notoginseng saponins inhibits ventricular remodeling after myocardial infarction in rats through regulating ATF3/MAP2K3/p38 MAPK and NF *κ* B pathway. *Chinese Journal of Integrative Medicine*.

[41] Ni S. Y., Zhong X. L., Li Z. H. (2020). Puerarin alleviates lipopolysaccharide-induced myocardial fibrosis by inhibiting PARP-1 to prevent HMGB1-mediated TLR4-NF-*κ*B signaling pathway. *Cardiovascular Toxicology*.

[42] Wang X. H., Li Z., Zang M. H., Yao T. B., Mao J. L., Pu J. (2020). Circulating primary bile acid is correlated with structural remodeling in atrial fibrillation. *Journal of Interventional Cardiac Electrophysiology: An International Journal of Arrhythmias And Pacing*.

[43] Corradi D., Callegari S., Benussi S. (2005). Myocyte changes and their left atrial distribution in patients with chronic atrial fibrillation related to mitral valve disease. *Human Pathology*.

[44] Patel D., Darki A., Hoppensteadt D. (2021). Biomarkers of thrombo-inflammatory responses in pulmonary embolism patients with pre-existing versus new-onset atrial fibrillation. *Clinical and Applied Thrombosis/Hemostasis: Official Journal of the International Academy of Clinical and Applied Thrombosis/Hemostasis*.

[45] Hohmann C., Pfister R., Mollenhauer M. (2020). Inflammatory cell infiltration in left atrial appendageal tissues of patients with atrial fibrillation and sinus rhythm. *Scientific Reports*.

[46] Yarmohammadi F., Hayes A. W., Karimi G. (2021). Possible protective effect of resolvin D1 on inflammation in atrial fibrillation: involvement of ER stress mediated the NLRP3 inflammasome pathway. *Naunyn-Schmiedeberg's Archives of Pharmacology*.

